# Analyzing the Chinese landscape in anti-diabetic drug research: leading knowledge production institutions and thematic communities

**DOI:** 10.1186/s13020-016-0084-y

**Published:** 2016-03-29

**Authors:** Junling Deng, Kaweng Sitou, Yongping Zhang, Ru Yan, Yuanjia Hu

**Affiliations:** State Key Laboratory of Quality Research in Chinese Medicine, Institute of Chinese Medical Sciences, University of Macau, Macao, China; Guiyang College of Traditional Chinese Medicine, Guiyang, Guizhou China

**Keywords:** Greater China, Anti-diabetes drug, Chinese medicines, Research collaboration networks, Network analysis

## Abstract

**Background:**

The discovery of anti-diabetic drugs is an active Chinese medicine research area. This study aims to map out anti-diabetic drug research in China using a network-based systemic approach based on co-authorship of academic publications. We focused on identifying leading knowledge production institutions, analyzing interactions among them, detecting communities with high internal associations, and exploring future research directions.

**Methods:**

Target articles published in 2009–2013 under the topic “diabetes” and subject category “pharmacology & pharmacy,” with “China,” “Taiwan,” “Hong Kong,” or “Macao” (or “Macau”) in the authors’ address field were retrieved from the science citation index expanded database and their bibliographic information (e.g., article title, authors, keywords, and authors’ affiliation addresses) analyzed. A social network approach was used to construct an institutional collaboration network based on co-publications. Gephi software was used to visualize the network and relationships among institutes were analyzed using centrality measurements. Thematic analysis based on article keywords and *R*_*sc*_ value was applied to reveal the research hotspots and directions of network communities.

**Results:**

The top 50 institutions were identified; these included Shanghai Jiao Tong University, National Taiwan University, Peking University, and China Pharmaceutical University. Institutes from Taiwan tended to cooperate with institutes outside Taiwan, but those from mainland China showed low interest in external collaboration. Fourteen thematic communities were detected with the Louvain algorithm and further labeled by their high-frequency and characteristic keywords, such as *Chinese medicines*, *diabetic complications*, *oxidative stress*, *pharmacokinetics*, and *insulin resistance*. The keyword *Chinese medicines* comprised a range of Chinese medicine-related topics, including *berberine*, *flavonoids*, *Astragaluspolysaccharide*, *emodin*, and *ginsenoside*. These keywords suggest potential fields for further anti-diabetic drug research. The correlation of −0.641 (*P* = 0.013) between degree centrality and the *R*_*sc*_ value of non-core keywords indicates that communities concentrating on rare research fields are usually isolated by others and have a lower chance of collaboration.

**Conclusion:**

With a better understanding of the Chinese landscape in anti-diabetic drug research, researchers and scholars looking for experts and institutions in a specific research area can rapidly spot their target community, then select the most appropriate potential collaborator and suggest preferential research directions for future studies.

**Electronic supplementary material:**

The online version of this article (doi:10.1186/s13020-016-0084-y) contains supplementary material, which is available to authorized users.

## Background

Diabetes mellitus (DM) is a chronic disease that is defined as a long period of having a high blood glucose level [1], which results in excessive thirst, frequent urination, and hunger. DM complications include ketoacidosis and non-ketotic hyperosmolar [2, 3]. According to the latest statistics of the International Diabetes Federation, the global prevalence of diabetes among adults aged between 20 and 79 years reached 8.3 % in 2013 [4]. A total of 382 million people worldwide are affected by the disease; of these, 80 % live in either mid- or low-income countries [4]. The number of people living with the disease worldwide is predicted to rise to 592 million by 2035 [4]. The prevalence of DM in China escalated from 0.9 % in 1980 to 11.6 % in 2010 [5, 6]. In 2013, diabetes caused 5.1 millions deaths, accounting for 8.39 % of global mortality [4]. The financial cost of diabetes in the U.S. exceeded $548 billion in 2013, accounting for 11 % of global expenditure on health care [4]. The rapidly growing prevalence of diabetes has placed an enormous burden on both social and economic development [4].

The discovery of anti-diabetic drugs has become an active research area in Chinese medicine [7]. Many institutes located in different regions of Greater China are currently engaged in the research and development (R&D) of anti-diabetic drugs. They cooperate on diabetes research projects and work concurrently on self-developed products. Some of these products, such as *Xiaoke wan* (消渴丸), *Yuquan pill* (玉泉片), and *Ginseng*–*milkvetch declining sugar granule* (參芪降糖顆粒) are among the most popular and well-known anti-diabetic drugs in China. Several studies have investigated the R&D of anti-diabetic drugs in China using bibliometrics to examine the distribution of publication years and journals, document types, keywords, citation counts, and journal impact factors [8, 9]. However, the network-based systematic method has not been applied to evaluate China’s pharmaceutical research in relation to diabetes, creating a gap in the understanding of the R&D landscape.

This study aims to map the R&D landscape of anti-diabetic drugs in Greater China (which includes mainland China, Taiwan, Hong Kong, and Macao) using a network-based systematic review. By analyzing co-authorship in the diabetic literature to include Chinese researchers published in international journals during the period 2009–2013, the active institutes in this field were identified, the relationship between research units was explored, communities with high internal associations were detected, and the interests of a research community were characterized.

## Methods

### Data

This systematic review was based on the web of science’s science citation index expanded (SCIE) database, a multidisciplinary index of more than 8500 peer-reviewed international journals [10]. Retrieved articles were those published during the period 2009–2013 under the topic “diabetes” and subject category “pharmacology & pharmacy,” with “China,” “Taiwan,” “Hong Kong,” or “Macao” (or “Macau”) in the authors’ address field.

**Fig. 1:**
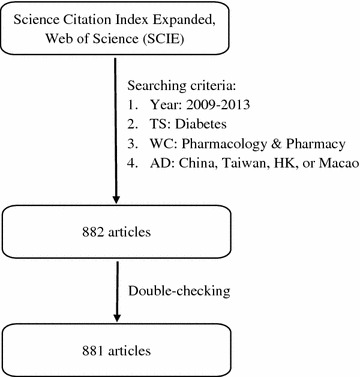
Flowchart of the inclusion and exclusion criteria. Combined search strategy: “AD = (China or Taiwan or Hong Kong or Macau or Macao or Hongkong) and WC = (Pharmacology & Pharmacy) and TS = (Diabetes) and Indexes = SCIE and Timespan = 2009–2013”. *AD* address, *TS* topic, *HK* Hong Kong, *WC* Web of Science Category which aims to narrow search result to some specific fields of study. Institutional information was obtained from the addresses provided by authors in the target articles, and was later standardized. Records of authors belonging to a non-Chinese institute were removed

Bibliographic information such as article title, authors, keywords, and authors’ affiliation addresses was extracted for analysis. Records of authors affiliated with non-Chinese institutes were eliminated. We standardized and combined authors and their affiliated organizations. The data retrieval process is illustrated in Fig. 1.

### Network visualization and analysis

The network was visualized using the Gephi software package (Version 0.8.2; The Gephi Consortium, Paris, France) and the relationships were analyzed to extract the associations between organizations. Each node in the network represents a research unit, and an edge between two nodes indicates the strength of co-authoring between these two units. Nodes were positioned using the Fruchterman–Reingold algorithm [11] in Gephi. The algorithm belongs to a class of algorithms known as force-directed algorithms; these are used to calculate layouts of simple undirected graphs. This method uses a physical analogy to determine the placement of network nodes by minimizing the energy of the system. Nodes behave like atomic particles; they exert repulsive and attractive forces on one another. Repulsive forces exist between all pairs of nodes (like charged particles repelling each other); edges between adjacent nodes cause attraction (like spring force). The position of all nodes continues to adjust until the system reaches its equilibrium state. Thus, the more connected organizations have higher attractive forces and are positioned at the center; in contrast, weaker nodes with less or no connections to others are found on the periphery of the map.

In this study, we applied four centrality metrics to identify the key institutes: degree centrality, weighted degree centrality, betweenness centrality, and closeness centrality.

Degree centrality measures centrality. This shows the number of ties associated with a node in an undirected graph and is reflected in node size. In our research, the degree centrality of an institute represents its number of collaborating organizations. Nodes with a high degree of centrality are usually found in the dense area of the network because they are working with many different research units.

Each node’s weighted degree was used to measure an institution’s actual performance in productivity and how active the organization was in the system. Weighted degree centrality corresponds to the sum of weighted edges connected to a vertex. In the present study, it signified an organization’s total number of co-publications.

Betweenness and closeness centrality describe the importance and role of a node in the system. Betweenness centrality measures how often a node appears on the shortest path between two nodes. In a collaboration network, some nodes do not interact directly; instead, they depend on an intermediary for communication. An intermediary with high betweenness functions as a “gatekeeper” to control the flow of interactions in the network. However, a high-betweenness node need not necessarily be one with a high degree centrality [12]. The closeness centrality of a vertex is the total geodesic distance between a vertex and all other vertices; it can be defined as how close an organization is to all others. A lower closeness value indicates that it is a more central node; that is, a node that can access or disseminate new information quicker than others [13, 14].

The above analysis focused mainly on absolute strength between nodes, but the relative strength between them was also considered. The Jaccard index is a statistic that shows how similar two sample sets are by calculating the ratio of intersection of the sets by the union of the sample sets [15]. Jaccard index values range from 0 to 1; a value closer to 1 indicates a higher mutual dependence between the two units. This value is defined as

$$J_{ij} = \frac{{N_{ij} }}{{\sum\nolimits_{j = 1}^{n} {N_{ij} } + \sum\nolimits_{i = 1}^{n} {N_{ij} } - N_{ij} }}\quad(i,j = 1, \ldots ,n;i \ne j)$$

where *N*_*ij*_ and *J*_*ij*_ stand for number of co-publications and relative strength of co-publication between institutions *i* and *j*, respectively. Referring to Scherngell and Hu’s use of this index [16], we used it to measure each institution’s level of mutual dependence on its collaborating pair for co-publication. We separately collected the top 50 institutional pairs measured by absolute (edge weight) and relative (Jaccard index) values. We selected the overlapping pairs between two rankings to determine the top institutional pairs in the Chinese anti-diabetic drug research network; this indicated those strongly connected institutional pairs that rely heavily on each other for publication.

### Community detection and thematic analysis

In the undirected network, some tightly interconnected nodes formed relatively stable community subnetworks. To quantify the notion of community, we used modularity, a measure that assigns a numeric value assessing how well a partition of the network nodes matches the informal notion of community. We used the Louvain algorithm, an efficient and widely used Gephi method to detect high-modularity communities, to determine the relevant communities in the network [17].

To characterize the themes of each identified community, the occurrence of each article keyword in the community was calculated and its relevance to that particular community was measured by its *R*_*sc*_ value, which is defined as *R*_*sc*_ = *f*_*sc*_*/f*_*s*_ [18]. This value is the ratio of occurrence of keyword S in community C’s articles to its occurrence in the article set as a whole. A high *R*_*sc*_ value indicates that a keyword’s occurrence in a specific community is relatively higher than in the whole system and is more significant to that community. Moreover, for each community, we categorized all non-core keywords as “Others” and calculated its *R*_*sc*_ value. The term “non-core keywords” describes keywords that appeared in the article set less than five times in total. Considering the linear and normally distributed characteristics of the data at the interval or ratio level, we then performed a Pearson’s correlation test (SPSS Statistics software, Version 20; IBM Corp., Armonk, NY, USA) between the *R*_*sc*_ value of “Others” and the degree centrality of that community to identify the relationship between research topics and collaboration opportunities of communities in the network.

## Results

### General description of data sample

Using the retrieval criteria mentioned in the Methods section, a total of 882 articles were retrieved. Excluding one correction article, we obtained 881 articles after the exclusion process. A majority of the sampled items were research articles (86.85 %); the rest comprised review articles (9.75 %), meeting abstracts (3.29 %), and proceedings papers (1.02 %). Most of these publications were funded by government or non-profit organizations. The top three funding sources were the National Natural Science Foundation of China (25.26 %), the National Basic Research Program (also called “973 Program”) (2.15 %), and the Fundamental Research Funds for the Central Universities (1.81 %). In this article set, the number of publications rose from 128 to 241 over the 5-year period of 2009–2013, representing a growth rate of 88.28 % or an annual rate of increase of 17.14 %. Increasing attention has been paid to anti-diabetic drug research in China and the discovery of new therapeutics for the disease has become increasingly important and urgent.

After standardizing and combining authors and their affiliated organizations, 430 research units were identified. Universities and their affiliated hospitals accounted for the largest proportion (48.84 %), followed by hospitals and clinics (24.88 %). There were fewer research centers and enterprises, accounting for only 16.98 and 9.30 %, respectively. Of all research units, 79.53 % were located in mainland China while 17.44 % were from Taiwan; the rest were from Hong Kong (2.56 %) and Macao (0.47 %).

### Institutional collaboration network

**Fig. 2:**
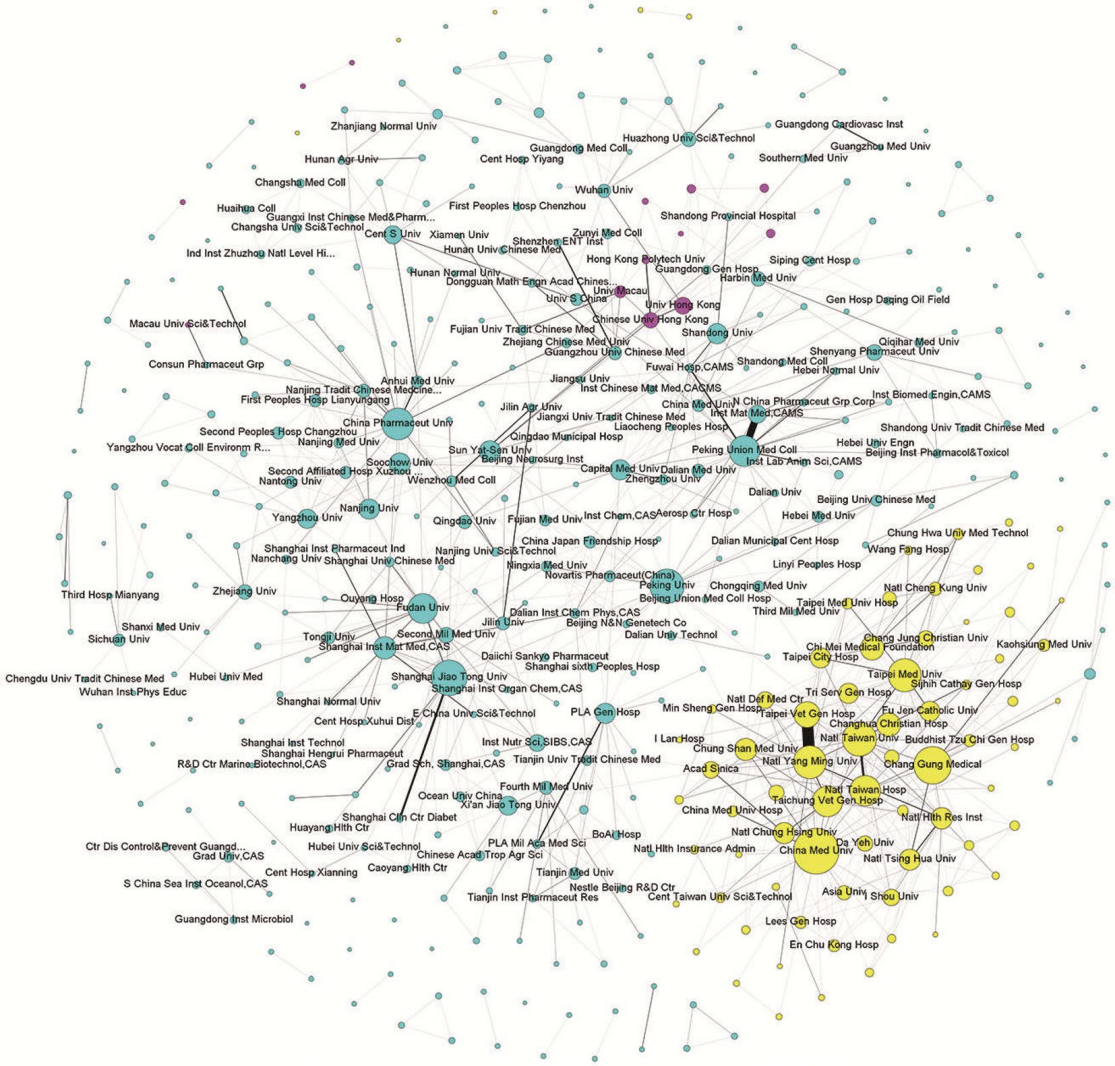
Institutional collaboration network of anti-diabetic drug research in China. This network is visualized and analyzed by using software Gephi, it comprises 430 *nodes* and 7673 *undirected weighted edges*. A *node* represents a research institution and node size depends on degree centrality while *edge* thickness between two nodes is determined by the number of co-publications between them. Research institutions from mainland China and Taiwan are represented in *cyan* and *yellow* respectively, and *magenta* represents those from both Hong Kong and Macao. Only the names of the more active nodes are shown here

Figure 2 illustrates the current collaborative relationship between Chinese institutes involved in the anti-diabetic drug research. There are 430 nodes on the map and 7673 undirected weighted edges, indicating that these 430 institutes have cooperated 7673 times. Node size depends on degree centrality and edge thickness between two nodes is determined by the number of co-publications between them [19]; more frequently collaborating institutional pairs are connected by a thicker edge.

In this figure, research units from mainland China and Taiwan are represented in cyan and yellow, respectively, and magenta represents those from both Hong Kong and Macao. Only the names of the more active nodes are shown here.

The structure of this network can be broadly divided into two parts. The left part is dominated by institutions from mainland China and those from Hong Kong and Macao, and the right part comprises mainly Taiwanese organizations. Although there were few connections between these two parts, each part contained abundant internal connections. The research units in Taiwan tended to collaborate with organizations from the same region, but less with those from mainland China, Hong Kong, or Macao.

### Leading knowledge producers

Table 1 shows the top 50 institutes in weighted degree, indicating the total number of an institute’s co-publications. In addition, the influence of a particular institute on others’ collaborative behavior was explored using betweenness and closeness centrality.

**Table 1:** Top 50 institutions ranked by weighted degree

Rank	Organizations	WD (LEC, %)	BC	CC
1	Shanghai Jiao Tong Univ	1756 (17.0)	8300	3.029
2	Natl Yang Ming Univ (TW)	1539 (63.7)	1222	3.841
3	China Pharmaceut Univ	1311 (22.8)	7233	3.441
4	Chang Gung Medical (TW)	1257 (14.6)	4579	3.809
5	Taipei Vet Gen Hosp (TW)	1112 (72.6)	623	3.859
6	Peking Union Med Coll	1101 (70.6)	6402	3.271
7	Fudan Univ	783 (27.7)	10,980	2.962
8	Inst Mat Med, CAS	750 (76.1)	500	3.894
9	Cent S Univ	725 (15.3)	3654	4.347
10	Chinese Univ Hong Kong (HK)	707 (20.9)	1251	3.718
11	Shanghai Inst Mat Med, CAS	690 (38.6)	3153	3.506
12	Jilin Univ	632 (21.5)	1662	4.365
13	Harbin Med Univ	597 (8.4)	242	3.994
14	Huazhong Univ Sci & Technol	566 (13.3)	1650	4.638
15	Peking Univ	556 (20.7)	21,524	2.729
16	Natl Taiwan Univ (TW)	502 (72.7)	9998	3.212
17	Fourth Mil Med Univ	482 (15.8)	1788	3.72
18	Wenzhou Med Coll	450 (26.2)	1456	3.703
19	Zhejiang Univ	397 (13.9)	1555	3.785
20	Natl Hlth Res Inst (TW)	395 (52.2)	568	3.988
21	Second Mil Med Univ	373 (27.6)	1475	3.588
22	Sichuan Univ	363 (11.8)	2018	5.829
23	Shanghai Clin Ctr Diabet	348 (38.8)	0	4.021
24	PLA Gen Hosp	324 (47.8)	4045	3.500
25	Natl Taiwan Hosp (TW)	309 (79.3)	642	3.888
26	Capital Med Univ	298 (35.2)	4486	3.194
27	Taipei Med Univ (TW)	281 (81.9)	1245	3.93
28	Univ S China	280 (25.4)	2700	4.079
29	Guangzhou Med Univ	278 (25.9)	0	6.147
30	Fuwai Hosp, CAMS	277 (50.9)	325	4.126
31	Third Mil Med Univ	267 (7.1)	339	4.374
32	Nanjing Tradit Chinese Medcine Univ	261 (39.5)	1282	4.076
33	China Med Univ (TW)	254 (78.3)	2810	3.932
34	Taichung Vet Gen Hosp (TW)	237 (88.6)	569	3.971
35	Anhui Med Univ	229 (52.0)	3161	3.641
36	Shenyang Pharmaceut Univ	208 (29.3)	2938	3.938
37	Hebei Med Univ	206 (13.6)	228	3.568
38	Guangzhou Univ Chinese Med	204 (71.6)	1468	3.694
39	Univ Hong Kong (HK)	198 (41.9)	3317	3.471
40	Nanjing Univ	196 (39.3)	1459	3.547
41	Wuhan Univ	188 (22.3)	2114	4.171
42	Nanjing Med Univ	188 (35.1)	113	4.197
43	Weifang Med Univ	187 (13.9)	0	1.000
44	Shanghai Univ Chinese Med	186 (33.3)	128	3.744
45	Shandong Univ	185 (61.6)	4118	3.815
46	Xuzhou Med Coll	184 (32.6)	339	4.429
47	Beijing Inst Technol	174 (29.9)	2	1.000
48	Sun Yat Sen Univ	166 (51.8)	4493	3.300
49	Jilin Agr Univ	162 (69.1)	815	4.371
50	Natl Chung Hsing Univ (TW)	159 (66.0)	364	3.926

Institutions based in Hong Kong and Taiwan are labelled HK and TW respectively, while others are from the mainland of China

*WD* weighted degree, *LEC* level of external collaborations, *BC* betweenness centrality, *CC* closeness centrality

The majority of mainland Chinese organizations on the list are outstanding academic institutes; these include the universities of project 985 and 211 and some of the country’s best-known hospitals and research centers. Most of these are located in Shanghai (18.42 %) and Beijing (15.79 %).

The weighted degree comprises two parts: internal and external degrees. The internal degree represents the number of articles authored by scholars within the same research unit. The external degree reflects the number of co-publications by authors from different institutes. As characterized by their high weighted degrees, Shanghai Jiao Tong University, National Yang Ming University, China Pharmaceutical University, Chang Gung Medical Foundation, Taipei Veterans General Hospital, and Peking Union Medical College are the key institutes in Greater China’s anti-diabetic drug research. Organizations that tend to collaborate externally rather than internally had a higher level of external collaboration (LEC). The average LEC value of Taiwanese institutes listed here was 66.99 %, indicating that they tended to cooperate more externally than internally. Conversely, mainland China’s LEC was much lower than that of Taiwan, at only 32.98 %, indicating a rather closed collaboration network. As an example, Shanghai Jiao Tong University ranked first in weighted degree but its LEC was only 17 %. In contrast, Peking University had the highest betweenness centrality in the whole system, acting as a gatekeeper of knowledge flow. Other organizations that showed strong betweenness performance include Fudan University, National Taiwan University, Shandong University, Sun Yat-Sen University, and the University of Hong Kong; all of these acted as information intermediaries located in different regions across Greater China.

Many high-betweenness organizations were also the ones with low closeness centrality, emphasizing their important position as knowledge brokers who could access and share new information more quickly than others (Table 1). Some organizations occupied the highest positions for co-publication numbers, betweenness, and closeness, demonstrating their extraordinary contributions to knowledge creation and dissemination. These institutions were Shanghai Jiao Tong University, China Pharmaceutical University, Peking Union Medical College Hospital, Fudan University, Peking University, National Taiwan University, University of Hong Kong, and Sun Yat-Sen University.

### Top institutional pairs

Institutional pairs that concurrently ranked in the top 50 in terms of edge weight and Jaccard index were chosen to identify the most frequently collaborating pairs with highest mutual dependence. Table 2 shows the 12 pairs meeting these criteria. Closed collaborators that depend heavily on each other included National Yang Ming University and Taipei Veterans General Hospital in Taiwan, and Peking Union Medicine College and the Institute of Materia Medica of the Chinese Academy of Medical Science in Beijing. The data suggested that some pairs showing strong, solid collaborations (83.33 %) were geographically adjacent.

**Table 2:** Top institutional pairs in Chinese diabetes drug research network

No.	Institutional pairs (region)		Co-publication frequency	Jaccard index (*J*_*ij*_; 0-1)
1	Natl Yang Ming Univ (Taiwan)	Taipei Vet Gen Hosp (Taiwan)	709	0.365
2	Peking Union Med Coll (Beijing)	Inst Mat Med, CAMS (Beijing)	446	0.317
3	Natl Taiwan Hosp (Taiwan)	Natl Taiwan Univ (Taiwan)	135	0.200
4	PLA Military Academy of Medical Sciences (Beijing)	PLA Gen Hosp (Beijing)	81	0.209
5	Guangzhou Univ Chinese Med (Guangdong)	Shenzhen ENT Inst (Guangdong)	72	0.290
6	Guangdong Cardiovasc Inst (Guangdong)	Guangzhou Med Univ (Guangdong)	64	0.204
7	Nanjing Mil Command, Nanjing Gen Hosp (Jiangsu)	Xuzhou Med College (Jiangsu)	50	0.249
8	Macau Univ Sci & Technol (Macao)	Consun Pharmaceut Grp (Guangdong)	32	0.457
9	Univ Macau (Macao)	Fujian Univ Tradit Chinese Med (Fujian)	32	0.182
10	Beijing BIT&GY Pharmaceut R&D (Beijing)	Beijing Inst Technol (Beijing)	32	0.174
11	Xiamen Univ (Fujian)	Fujian Univ Tradit Chinese Med (Fujian)	27	0.197
12	State Adm Tradit Chinese Med (Beijing)	Hunan Agr Univ (Hunan)	25	0.379

Institution’s regional location is the provincial-level administrative divisions of China

### Thematic communities

**Fig. 3:**
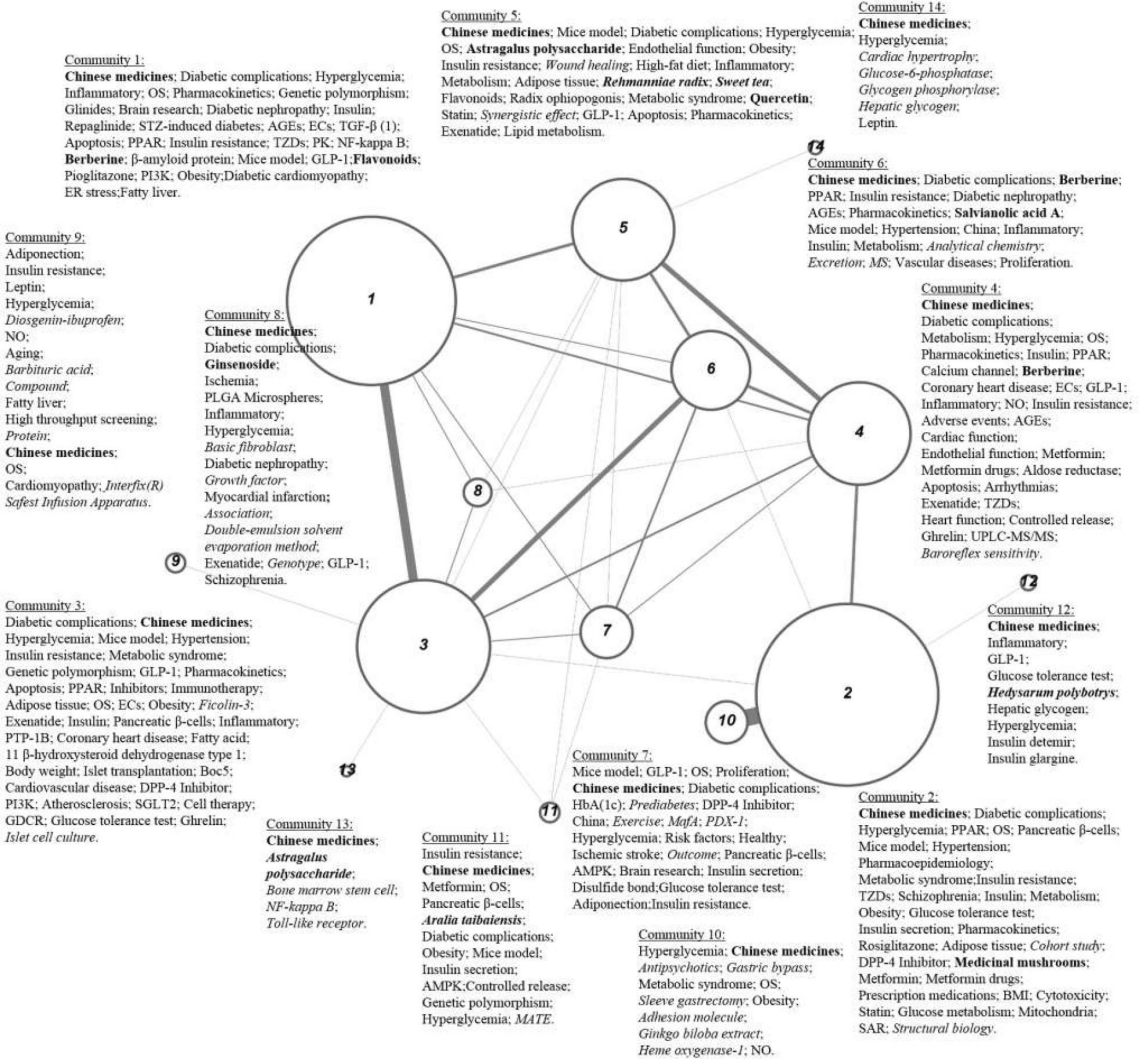
Thematic community network of diabetes drug research in greater China. This figure consists of 14 communities which are shown as *nodes* and labelled by their high frequency keywords in which *italic* *words* are research topics more relevant to a given community than to the others and those letters in *bold* are more related to Chinese medicines. Community size increases with number of articles, and *edge* thickness between any two nodes corresponds to the frequency of co-publications involving authors from those two communities. These subnetworks are labelled in descending order of number of institutes

The basic elements of an institutional collaboration network are the nodes and subgroups of the network, with various subgroups combining to form a complex institution network. As shown in Fig. 3, closely collaborating organizations were clustered into various community subnetworks. The 14 major subnetworks in this system were labeled in descending order of number of institutes. For example, community 1 was the largest community, with 71 organizations, whereas community 14 was the smallest, with only three organizations. Community size increases with number of articles, and edge thickness between any two nodes corresponds to the frequency of co-publications involving authors from those two communities. Table 3 contains additional information about the 14 communities.

**Table 3:** Detailed information of 14 communities

Community codes	Number of institutes	Number of articles	Frequency of co-publications
1	71	275	1159
2	64	285	769
3	44	197	881
4	34	152	618
5	33	151	564
6	30	117	413
7	21	77	252
8	11	41	215
9	8	28	122
10	7	52	234
11	6	29	137
12	6	12	34
13	3	5	22
14	3	9	65

Community size increases with number of articles, and the subnetworks are labelled in descending order of number of institutes. The edge thickness between any two nodes reflects the frequency of co-publications involving authors from those two communities

The internal structure mining of communities revealed geographic features. For community 2, only one out of the 64 institutes was outside Taiwan, indicating that this community was dominated by Taiwanese organizations that seldom communicate with mainland China, Hong Kong, or Macao. Community 3 was composed mainly of outstanding institutes from Shanghai, such as Shanghai Jiao Tong University, Fudan University, Shanghai Institute of Materia Medical of Chinese Academy of Sciences, and The Second Military Medical University. In contrast, all six research units in community 11 were found in Northwest China, including five from Shaanxi Province and one from the Xinjiang Uygur Autonomous Region.

The research interests of communities were identified by calculating the *R*_*sc*_ value of article keywords. For each community, keywords were arranged in descending order of frequency; those accounting for the top 50 % of cumulative frequency were shown and those in bold and in italic were the ones with top 10 *R*_*sc*_ values and more related to Chinese medicines respectively. As an example, community 2 focuses not only on *Chinese medicines*, *diabetic complications*, and *hyperglycemia*, but also on *cohort study* and *structural biology*.

All keywords appearing in the system were standardized for statistical analysis. Those occurring more than five times were classified as core keywords and the rest were classified as non-core keywords. The top core keywords were *Chinese medicines*, *diabetic complications*, *oxidative stress*, *hyperglycemia*, *insulin resistance*, *mice model*, *inflammatory*, *pharmacokinetics*, and *GLP*-*1*. The most frequent *Chinese medicines* keywords included *berberine*, *flavonoids*, *Astragaluspolysaccharide*, *emodin*, *ginsenoside*, *quercetin*, *curcumin*, *Ganoderma lucidum*, and *resveratrol*. Table 4 shows the most frequently used keywords in Chinese medicine anti-diabetes research; these are categorized into “Herbs” and “Compounds.” More than three research articles focused on “Herbs” and more than five articles focused on “Compounds.”

**Table 4:** Frequent keywords relevant to Chinese medicines

Herbs (frequency ≥ 3)		Compounds (frequency ≥ 5)	
Items (Chinese)	Frequency	Items (Chinese)	Frequency
*Coptidis Rhizoma (黃連)*	27	*Berberine (黃連素)*	27
*Medicinal mushrooms (藥用蘑菇)*	8	*Flavonoids (黃酮類化合物)*	25
*Ginseng (人參)*	7	*Astragalus polysaccharide (黃芪多醣)*	12
*Ganoderma lucidum (靈芝)*	5	*Polysaccharide (多聚糖)*	10
*Potentilla discolor bunge (翻白草)*	4	*Emodin (大黃素)*	8
*Radix ophiopogonis (麥冬)*	4	*Ginsenoside (人參皂苷)*	7
*Salvia miltiorrhiza (丹參)*	4	*Quercetin (槲皮素)*	7
*Buckwheat (蕎麥)*	3	*Curcumin (薑黃素)*	6
*Cinnamon (肉桂)*	3	*Salvianolic acid A (丹酚酸A)*	6
*Folium Eriobotryae (枇杷葉)*	3	*Triterpenes (三萜類化合物)*	6
*Paeonia lactiflora (芍藥)*	3	*Resveratrol (白藜蘆醇)*	5
*Selaginella tamariscina (卷柏)*	3		
*Swertia (獐牙菜)*	3		

The Pearson’s test of correlations between degree centrality and the *R*_*sc*_ value of non-core keywords across communities produced a negative correlation coefficient of −0.641 (*P* = 0.013), indicating that when a community focuses on a research topic that is of common interest to all communities, it can easily build up its collaboration network. However, communities that concentrate on rare research fields are usually isolated by others and have a lower chance of collaboration. For more details regarding the collaboration network and thematic communities, please refer to the Additional files 1, 2, 3, 4.

## Discussion

In the present study, the sample data included 881 scientific articles published by 430 Chinese institutes over the 5-year period of 2009–2013. This study identified the most productive institutes and leading academic communities in anti-diabetic drug development.

The average LEC of mainland China’s organizations was only half that of Taiwan, indicating that institutes in China were less dependent on external collaboration. This might be explained by a number of factors: (1) the organization had sufficient resources to meet the needs of research projects; (2) there might be no adequate communication platform for knowledge sharing; and (3) geographic distance resulted in a negative impact on information flow between institutes. In contrast, although Taiwan exhibited a high LEC, it was rather disconnected from organizations in mainland China, Hong Kong, and Macao.

The network visualized data in this article may help researchers and scholars to navigate in this active research area and to find potential collaborators specializing in a particular research area. For instance, a researcher affiliated with Jilin University (a member of community 8) who specializes in the research of *ginsenoside*, is planning to explore the therapeutic effect of a combination of *ginsenoside* and *medicinal mushrooms* on diabetic complications. In Fig. 3, community 2 was the only group to focus on *medicinal mushrooms* research; therefore, the researcher could probably identify potential collaborators from this community. Knowledge of the list of institutes that belong to community 2, and with the help of the co-authorship network, the researcher could select the most appropriate expert for research collaboration based on centrality measurements. In this case, the researcher might approach China Medical University in Taiwan, as it ranked at the top in the three aspects of centrality measurements among institutes studying *medicinal mushrooms*. This kind of research collaboration does not simply represent the building of a new partnership between two researchers or institutions, but also fosters knowledge exchange across various diabetes research communities in China and further stimulates innovation and productivity.

In addition, each community studied here is labeled with a list of keywords, covering a diverse range of topics that included *herbology*, *chemical component*, *molecular biology*, *clinical medicine*, *pharmacokinetics*, *therapeutic drugs*, and *animal experimental models*. *Chinese medicines*, *diabetic complications*, *oxidative stress*, *hyperglycemia*, and *insulin resistance* were common topics that could be found on the keyword list across different communities, indicating their significance in the field. Only minimal adverse effects were reported for Chinese medicines used in treating type 2 DM, indicating certain advantages in the prevention of diabetes and delay of its complications [7]. Studying the role of oxidative stress in the pathogenesis of diabetes provides a theoretical basis for the prevention and treatment of the disease [20], as oxidative stress could be a major cause of the development of type 2 DM [21]. Furthermore, *Chinese medicines* in combination with insulin exhibited better clinical effect in the treatment of gestational diabetes [22]. Similarly, *berberine* exhibited beneficial anti-inflammation effects, indicating that it could be a potential therapeutic drug in type 1 DM treatment [23].

Each of the 14 communities in the studied system has its own area of expertise, which is usually a non-mainstream topic. Community 2, a subnetwork primarily composed of Taiwanese organizations, focuses on *cohort study* and *structural biology* while Community 3 is a Shanghai-based group that specializes in *ficolin*-*3* and *islet cell culture*. Community 5 focuses on *wound healing*, *radix Rehmanniae*, *sweet tea*, and *synergistic effect*; most of its constituent organizations are from Guangdong Province, Hong Kong, and Macao. Finally, the Beijing-dominated Community 6 concentrates on *analytical chemistry*, *excretion*, and *mass spectrometry*.

The collaboration opportunities available to a community are strongly affected by the level of interest in its research topics. Communities dedicated to less-studied research areas usually have more difficulty in identifying suitable collaborators. This community thematic analysis provides a reference for researchers and institutes looking for potential collaborators based on research direction.

Researchers would find it easier to create research partnerships if the key researchers’ information was also directly available. However, this study explored the research landscape of Chinese anti-diabetic drugs only at an institutional level. It is more difficult to study research at a personal level because of data sensitivity and privacy protection, although we did have available personal publication data to identify the most prolific or influential authors in specific research areas, units, or communities in the system. Those researchers interested in author-related information can consult the list of articles for each thematic community in Additional file 4. For instance, a researcher searching for information related to *berberine* can search for the keyword *berberine* in Additional file 3, which provides the “Article No.” that contains this keyword. As each article carries a unique article number, this code can directly identify the article title, authors of the paper, authors’ affiliations and geographic region, and the thematic community to which the article belongs.

Because of the need for a homogeneous sample composed of cutting-edge research articles and regional biases, we chose to use just the SCIE data source and to not include Chinese literature indexed in CNKI and other Chinese databases. In using co-authorship academic publication data, this study mainly focuses on basic research and is limited to academic collaborations. A similar exercise for applied and competitive research would be an important addition to the current study and could, for instance, focus on collaboration in patenting and R&D projects. In addition, the arbitrary selection of keywords and the lack of keywords in a small proportion of sample articles may have caused potential bias in the thematic analysis. Future studies could construct a more complex and more influential collaboration network by expanding sample size or time span, or by widening the scope from domestic to international organizations. A dynamic network analysis could also be introduced to investigate how the network evolves over time.

## Conclusion

By characterizing communities and noting their research themes, the results of this study could provide guidance to researchers seeking potential collaborators and could suggest a precise research direction for future studies.

## Additional files

10.1186/s13020-016-0084-y Information of 430 institutes. The region information and all centrality measurements of 430 institutes are clearly presented.
10.1186/s13020-016-0084-y Tabular data of Fig. 3. All keywords in Fig. 3 are numbered by their frequency in each community, and the words in italics are research topic relevant to a given community than to other keywords and the bold words are more related to Chinese medicines.
10.1186/s13020-016-0084-y All keywords in each article. The keywords in different articles are listed, and their Chinese translation are also presented for reference.
10.1186/s13020-016-0084-y Details of 881 publications in 14 thematic communities. This document provides more detailed information of all articles in 14 thematic communities, it contains: article title, author, organization, geographic region and thematic community.

## Abbreviations

CAMS: Chinese Academy of Medical Science
CAS: Chinese Academy of Sciences
DM: diabetes mellitus
GDM: gestational diabetes mellitus
GLP-1: glucagon-like peptide-1
IDF: International Diabetes Federation
IMM: Institute of Materia Medica
LEC: level of external collaboration
MS: mass spectrum
NO: nitric oxide
OS: oxidative stress
R&D: Research and development
SCIE: science citation index expanded
T1DM: type 1 diabetes mellitus
T2DM: type 2 diabetes mellitus
